# Treadmill perturbation-based balance training to prevent unrecovered falls in fall-prone older adults with and without cognitive impairment: protocol for the multi-center randomized controlled TRAIL study

**DOI:** 10.1186/s12877-025-06599-w

**Published:** 2025-12-23

**Authors:** J Koschate-Storm, C Werner, J Bartel, JM Bauer, C Becker, S Drefs, N El-Seoud, C Giehl, M Hackbarth, N Hezel, J Klenk, U Trampisch, R Wirth, T Zieschang, M Schwenk

**Affiliations:** 1 Geriatric Medicine, University of Oldenburg, Oldenburg, Germany; 2 Geriatric Center, Medical Faculty Heidelberg, Heidelberg University, Heidelberg, Germany; 3 Marien Hospital Herne, University Hospital Ruhr-University Bochum, Herne, Germany; 4 Institute of Epidemiology and Medical Biometry, Ulm University, Ulm, Germany; 5 Department of Sport Science, Human Performance Research Centre, University of Konstanz, Konstanz, Germany

**Keywords:** Fall prevention, Reactive balance, Physical activity, Outpatient care, Cognition

## Abstract

**Background:**

Approximately one-third of older adults fall each year, most often due to slips or trips. Fall incidence is even higher in those with cognitive impairment (CI). Among older adults who fall, about 50% are unable to get up without assistance. Such unrecovered falls are particularly critical, as they are linked to prolonged lying periods, an increased risk of medical complications, and mortality. As unrecovered falls require third-party assistance, they offer an opportunity to incorporate proxy information into fall reporting to reduce recall bias. Perturbation-based balance training (PBT) aims to improve recovery reactions in response to balance disturbances such as slips and trips and thereby prevent falls. This task-specific approach has shown promise in reducing falls in low-risk older adults. However, its efficacy in high-risk populations, especially in participants with CI, remains largely unknown. The primary aim of the TRAIL study is to evaluate the efficacy of treadmill PBT in reducing unrecovered falls among fall-prone older adults with and without CI.

**Methods:**

In this multi-center, assessor-blinded, randomized controlled parallel-group trial, 396 older adults (≥ 70 years) at risk of falling (≥ 40% prospective fall risk, Timed Up and Go ≥ 10 s) will be assigned (1:1) to receive nine sessions of either treadmill PBT or conventional treadmill training (CTT) over three weeks. The primary outcome is the incidence of unrecovered falls within twelve months post-intervention, tracked via monthly fall calendars, phone interviews, proactive reporting, and proxy information. Secondary outcomes include other fall-related outcomes (e.g. total falls, injurious falls, falls per physical activity), physical capacity and activity, psychological status, and cognitive functioning. Assessments will be conducted at baseline, post-intervention, as well as six and twelve months after the intervention. Primary analyses will follow the intention-to-treat principle.

**Discussion:**

Treadmill PBT is expected to reduce unrecovered falls by ≥ 50% over twelve months, compared to CTT. If effective, the low-volume PBT approach can serve as an important treatment option for the rapidly growing group of fall-prone older adults, especially those with CI, for whom evidence-based strategies for fall prevention remain limited.

**Trial registration:**

Prospectively registered at ClinicalTrials.gov (NCT06652828). First posted: 2024-10-22, last update posted: 2025-05-14.

**Supplementary Information:**

The online version contains supplementary material available at 10.1186/s12877-025-06599-w.

## Background

In Germany, 23.8% of adults aged 65 years and older, and 33.5% of those aged 80 years and above, reported at least one fall in 2022 [1]. This aligns with global estimates of the World Health Organization [2]. Even more concerning are the data for older adults with cognitive impairment (CI). An annual fall incidence of 39.6% was recently reported, based on data of ‘The Norwegian Registry of Persons Assessed for Cognitive Symptoms’ [3].

The consequences of falls can be serious, even in the absence of injury, as they often lead to concerns about falling that frequently reduce social participation and quality of life, increase sedentary behavior, and ultimately contribute to a vicious circle that increases the risk of losing independence [4].

The latest update of the 2019 Cochrane review [5] and other systematic reviews [6, 7] conclude that physical exercise has the potential to reduce the number of falls in older adults by approximately 20–25%. The World Falls Guideline recommends that exercise programs for older adults include balance and functional training at least three times per week, with progressively increasing intensity over a minimum of 12 weeks, and, when feasible, incorporate progressive resistance training [8]. However, since many older adults lead physically inactive lifestyles [9], the implementation and acceptance of such high-frequency, long-duration exercise programs may be limited. Furthermore, two pragmatic studies conducted for the first time with sufficient statistical power (*n* >2,000) to study fall-related injuries as primary outcome, did not find significant benefits of multifactorial fall prevention interventions that include such exercise programs [10, 11]. One possible reason for these limited effects is that conventional balance, functional, and resistance exercises may not be sufficiently task-specific to train preventive mechanism underlying most falls. This highlights the need for better exercise training strategies in fall prevention [12].

Slipping and tripping are the most common causes of falls and fall-related injuries among older adults [13, 14], and reactive balance, the ability to respond effectively to balance disturbances (e.g. slips, trips) in order to avoid a loss of balance and falling, has been identified as a significant risk factor for falls in this population [15]. Reactive balance can be trained via perturbation-based balance training (PBT), defined as ‘*balance training that uses repeated*,* externally applied mechanical perturbations to trigger rapid reactions to regain postural stability in a safe and controlled environment’* [16]. Such task-specific reactive balance exercise has been identified as the most promising for improving reactive balance in older adults [17].

An early study applying PBT stated an impressive reduction in falls of about 50% over a 12-month follow-up period after a single session of overground slip perturbations [18], which is approximately twice the effect reported for conventional physical exercise programs, while requiring considerably less training volume [19–21]. The most recent systematic review on the effects of PBT reported a 39% reduction in fall rates compared to controls among older adults and certain patient groups (e.g., stroke, Parkinson’s disease, chronic obstructive pulmonary disease) [22]. Subsequent original studies have shown mixed results. Nørgaard et al. [23] observed a statistically non-significant 22% reduction of daiy-life fall rate after twelve months in community-dwelling healthy older adults who completed three sessions of treadmill slip and trip perturbations within one week, along with a booster session after six months, compared with conventional treadmill training (CTT). In contrast, Rieger et al. [24] reported significantly fewer daily-life falls in community-dwelling healthy older adults after dual-task treadmill PBT, delivered twice per week over four weeks, compared to dual-task treadmill training without perturbations. The number of daily-life falls decreased by 64% from baseline in the treadmill PBT group.

To date, the efficacy of PBT in reducing falls has not been documented in higher-risk populations, especially those with CI, despite CI being a well-known risk factor for falls [25–27]. In fact, recent PBT studies often excluded participants with CI [18, 24, 28–30]. A recent pilot study evaluated treadmill PBT in pre-frail and frail older adults with and without CI [31]. Although its feasibility and effectiveness in improving physical capacity were demonstrated [31, 32], the study did not assess daily-life fall outcomes.

The total number of self-reported falls is the most established primary outcome in fall prevention trials [5], but is prone to recall biases, especially in populations with CI [33, 34]. Injurious falls, while clinically highly relevant and potentially less affected by recall biases [35, 36], occur less frequently, requiring larger sample sizes to provide sufficient statistical power for detecting intervention effects. Unrecovered falls, where an individual is unable to get up without assistance [37], may offer favorable balance between clinical relevance and feasibility of fall data collection. They occur more frequently than injurious falls, yet still representing particularly critical events, as they often result in prolonged lying periods that can lead to serious medical complications [38–40], and increase the risk of hospital admission, institutionalization, and mortality [19, 39, 41]. Beyond individual health risks, unrecovered falls place significant strain on emergency and prehospital care systems. Over 80% of older adults activating a personal alarm after a fall were still on the ground upon paramedic arrival [42], and 72% of non-emergent lift-assist calls, where inividuals need help standing without medical intervention, are fall-related, accounting for a substantial share of ambulance resources [43]. About half of community-dwelling older adults who fall cannot get up without assistance [37, 39], with rates rising to 80% among nonagenarians [19]. This inability has also been assoiated with lower cognitive functioning [19, 27]. The higher incidence rate of unrecovered falls may increase statistical power and allow for smaller sample sizes in fall prevention trials. In addition, unrecovered falls may offer the opportunity to use information from third-party assistance to improve fall reporting accuracy, which is particularly important in populations with CI. Their distressing nature and need for external help may also make unrecovered falls more salient and thus better recalled by the persons themselves.

The primary aim of the TRAIL study is to evaluate the efficacy of treadmill PBT in reducing unrecovered falls in fall-prone older adults with and without CI. The main hypothesis is that treadmill PBT will result in a significantly lower incidence of unrecovered falls over a 12-month follow-up period compared to CTT.

## Methods

### Study design

The TRAIL study (Training at the limit of balance control on a perturbation treadmill to prevent unrecovered falls in geriatric patients with and without cognitive impairment) is designed as a multicenter, randomized (1:1), controlled, parallel-group intervention trial. A total of 396 participants will be recruited at three German study sites: Oldenburg, Bochum-Herne, and Heidelberg (*n* = 132 at each site). The study protocol is reported according to the SPIRIT checklist for study protocols (Standard Protocol Items: Recommendations for Interventional Trials) and was registered prospectively at ClinicalTrials.gov (NCT06652828).

### Participants and recruitment

Eligible individuals are included if they are 70 years and older, have a prospective fall risk of ≥ 40% as determined via the Fall Risk Assessment Tool [44], and are able to walk ≥ 70 m in the 2-minute walk test (2MWT) without the use of walking aids [45, 46]. Individuals are excluded if they have a Montreal Cognitive Assessment (MoCA) score < 10 pts [47] or Mini-Mental State Examination (MMSE) score < 17 pts [48], a Timed up and Go (TUG) < 10 s without a walking aid [49], body weight >135 kg or body height >185 cm, leg paralysis, leg amputation, gait relevant foot drop paresis, functional blindness, Parkinson’s disease with Hoehn and Yahr stage >3 [50], osteosynthesis or joint replacement of lower extremities within the past six weeks, prior experience in PBT, life expectancy of less than 12 months, instable or severe illness, and inability to follow testing or training instructions, or to communicate verbally.

Participants are recruited during their stay in hospitals, inpatient and outpatient rehabilitation centers, and day clinics, as well as from the community. In the clinical setting, potential participants are informed about the study, screened for eligibility, and asked to provide informed consent during their stay. Community-based recruitment is conducted via advertisements (e.g., local newspaper articles, posters, and flyers at senior centers) and by contacting individuals from patient registries of the study centers. The eligibility of these community-recruited participants is screened using a combination of telephone interviews and on-site screening (2MWT, TUG, MoCA, or MMSE).

Consent forms are available in plain language. If a participant has a legal guardian, the guardian must sign the respective consent form on the participant’s behalf. Participants are also asked to name one or two proxies to be contacted by the study team to provide additional information on unrecovered fall events or confirm the plausibility of fall reports, in cases of missing data, implausible or contradictory reports, or unavailability of the participant. The proxies are contacted by the study team and will also provide written informed consent prior to participation. Participation of a proxy is not obligatory for the participant’s enrollment in the study. Travel costs of the participants will be reimbursed, and transportation to the study center via taxi will be offered.

**Fig. 1:**
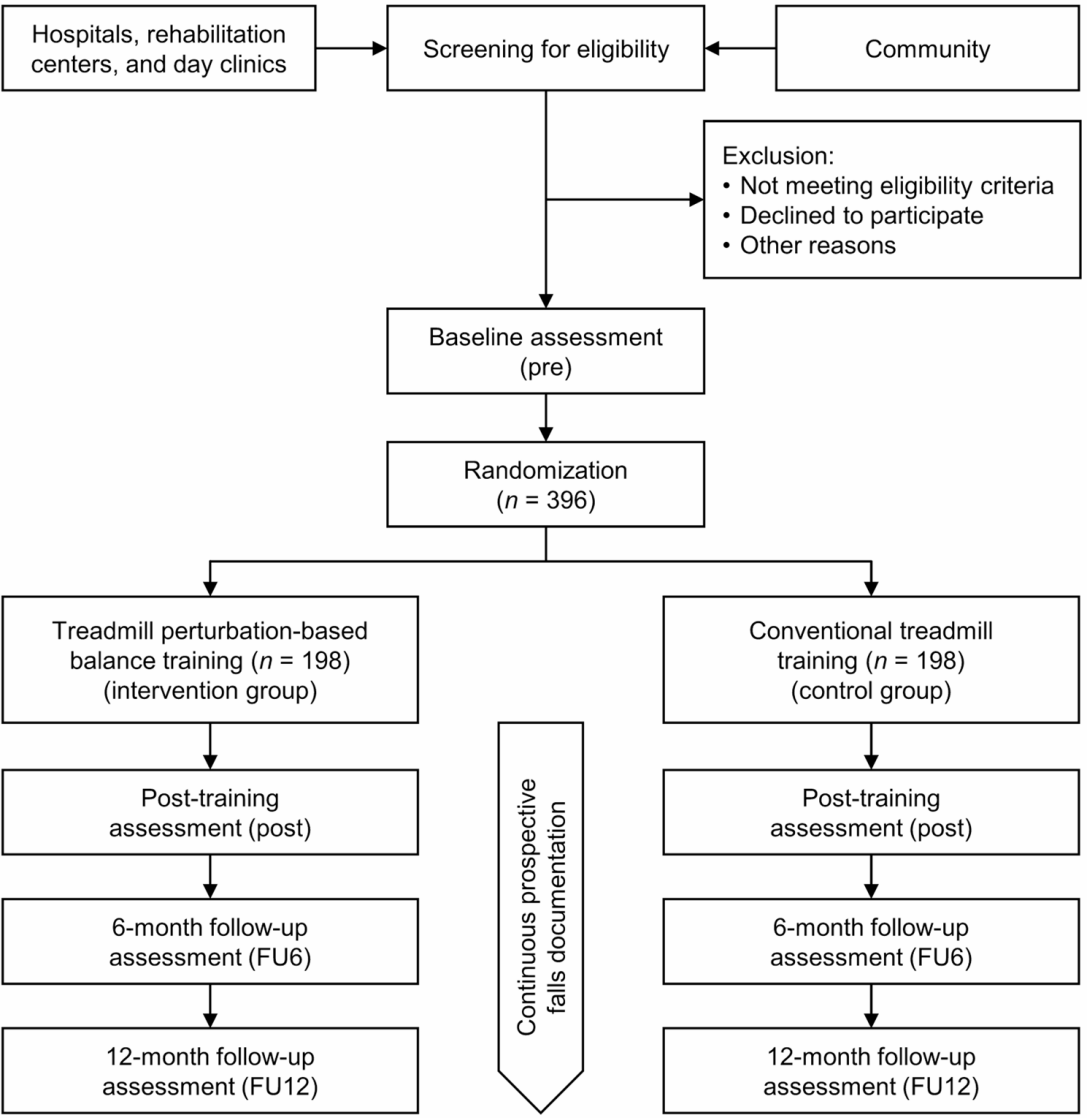
Flowchart of the participants in the TRAIL study

The screening, recruitment, enrollment and participation process throughout the study is shown in Fig. 1.

### Randomization and blinding

Participants will be randomized to the control (CTT) or intervention group (treadmill PBT) after the baseline assessment. Block randomization with variable block sizes and a 1:1 allocation ratio, stratified by study site will be used. The randomization list will be generated by the Institute of Epidemiology and Medical Biometry of Ulm University. Assessors will be blinded to group allocation; however, blinding is not possible for the participants and the training staff. Participants will be informed about receiving treadmill PBT or CTT by the trainer. Data that reveals group allocation, such as the acceptability of the treadmill PBT or dynamic reactive balance at training sessions three and nine, will be assessed by unblinded training staff. At the end of the study, after database lock, the study statistician will be unblinded to conduct the final analysis.

### Interventions

All participants will receive nine training sessions on a perturbation treadmill (BalanceTutor, MediTouch, Netanya, Israel) over a period of three weeks, with three sessions per week. In case of illness, scheduling conflicts or organizational issues, the training period may be extended to a maximum of six weeks to ensure completion of all nine sessions. Each training session will consist of a 2.5-min warm up, followed by five training blocks of 2.5 min each. During each warm-up, the individually preferred walking speed is determined and subsequently used for all subsequent training blocks in that session. This speed is identified by starting at 50% of the participant’s habitual overground walking speed (derived from the 2MWT at baseline assessment), then gradually increasing and decreasing the treadmill speed to determine the upper and lower bounds of comfortable walking. The average of these bounds is defined as the individually preferred walking speed [23, 28].

Participants in the intervention group (treadmill PBT) will receive surface perturbations while walking on the treadmill, whereas those in the control group (CTT) will walk without perturbations. Training duration and individual walking pace will be the same in both groups. Each session in both the control and intervention groups will include about 15 min of exercise and a total of about 10 min of breaks. All participants will be secured by an overhead safety harness during treadmill walking. Training sessions will be conducted by specifically trained sports scientists and physiotherapists. A detailed description of the two interventions is provided in the TIDieR (Template for Intervention Description and Replication) checklist in Supplementary Table 1.

#### Treadmill perturbation-based balance training

The treadmill PBT intervention has been specifically developed for the target group of fall-prone older adults, based on previous experience and studies conducted by members of the TRAIL research group [28, 31, 51]. Each of the nine treadmill PBT sessions is structured with specific training goals that are layered and each one adding progressively to the previous one (Table 1). The treadmill PBT intervention starts with familiarization and fostering confidence, walking on the treadmill (sessions 1–2), followed by a phase of reduced predictability and increased intensity of perturbations (sessions 3–5), progressing to varied perturbation intensities (sessions 6–7), and training combined with simultaneous presentation of a video during walking (session 8–9). Given the participants’ functional and cognitive impairments, the initial sessions present a lower level of challenge. The low-threshold initiation of the treadmill PBT intervention is specifically designed to increase perturbation tolerability and mitigate anxiety, which may arise, particularly at the beginning of the intervention [16, 52]. Perturbations will initially be predictable, with both timing and direction, announced in advance, and will be applied exclusively in the anterior-posterior (AP) direction (session 1). Mediolateral (ML) perturbations will be introduced in session 2, as they are more challenging [53] and may lead to higher levels of stress and associated anxiety [54]. Once participants are comfortable with treadmill PBT, the intensity will be gradually increased, and unannounced perturbations will be introduced (session 3–5) as a key feature of PBT [16]. In sessions six to nine, the intensity of the perturbations will vary both within training blocks and between the individual perturbations. These variations are intended to facilitate overlearning and optimize training effects [55].

The treadmill PBT intervention is designed to individually challenge each participant at the limits of their balance control, promoting the development of balance recovery strategies. Therefore, training progression will be controlled via trainer and participant feedback, using a 5-point Likert scale, asking about individually perceived anxiety (not at all, just a little, mildly, moderately, extremely) and difficulty (1 = easy, 2 = fairly easy, 3 = challenging, 4 = very challenging, 5 = too hard) at the end of each training block [56]. The first two sessions will include perturbations with a difficulty level of ≤ 3 points on the 5-point Likert scale. Starting from session three, the difficulty level will be increased to 3–4 points, based on the trainers’ judgement. The predictability of perturbation directions will decrease throughout the intervention: in session one, two directions (forward, backward) and in session two four directions (forward, backward, left, right) will be applied, and timing and direction of the perturbation will be announced. During the third session, the direction of the perturbations will be announced at the beginning of each block. From session four onwards, all perturbation directions will be applied unpredictable and unannounced. From session eight onward, walk-through landscape videos will be shown during the blocks to increase task difficulty through standardized distraction, and to make the training more engaging.

All trainers across the three intervention sites will receive comprehensive training in the structured treadmill PBT protocol, based on a standardized trainer manual. Additionally, trainers will be regularly supervised via videoconferences to ensure intervention fidelity.

**Table 1:** Description of the aims and structured content of the nine perturbation training sessions

Session No	Session aim	Session content			
		Perturbation number	Perturbation directions	Perturbation predictability	Perturbation intensity (level of difficulty^a^)
1	Treadmill familiarization	24	AP (forward, backward)	Direction and timing announced	≤ 3
2	Gain confidence on the treadmill	32	ML (left, right) AP (forward, backward)	Direction and timing announced	≤ 3
3	Increase in perturbation intensity and reduction of predictability	40	ML (left, right) AP (forward, backward)	Direction announced	3–4
4	Increase in perturbation intensity and reduction of predictability	40	ML (left, right) AP (forward, backward)	Unannounced	3–4
5	Increase in perturbation intensity	40	ML (left, right) AP (forward, backward)	Unannounced	3–4
6	Contrast in perturbation intensity between blocks	40	ML (left, right) AP (forward, backward)	Unannounced	3–4 2–3
7	Contrast in perturbation intensity within blocks	40	ML (left, right) AP (forward, backward)	Unannounced	3–4 2–3
8	Video during perturbed walking, contrast in intensity between blocks	40	ML (left, right) AP (forward, backward)	Unannounced	3–4 2–3
9	Video during perturbed walking, contrast in intensity during blocks	40	ML (left, right) AP (forward, backward)	Unannounced	3–4 2–3

*AP* anterior-posterior, *ML* mediolateral

^a^5-point Likert scale (1 = easy, 2 = fairly easy, 3 = challenging, 4 = very challenging, 5 = too hard)

#### Conventional treadmill training

Participants in the CTT group will walk at their individually preferred walking speed throughout the nine sessions, with this speed redetermined during the 2.5 min warm-up at the beginning of each session, as in the treadmill PBT group. Videos will likewise be shown in sessions eight and nine.

### Outcomes

Outcome measures will be assessed before the intervention (pre; at maximum two weeks after the screening), within two weeks after the intervention (post), and six (FU6), and twelve months (FU12) post-intervention. Falls will be recorded continuously throughout the entire study period. An overview of the outcome measures, screening parameters, and descriptive variables across the study timeline is given in Table 2.

**Table 2:**
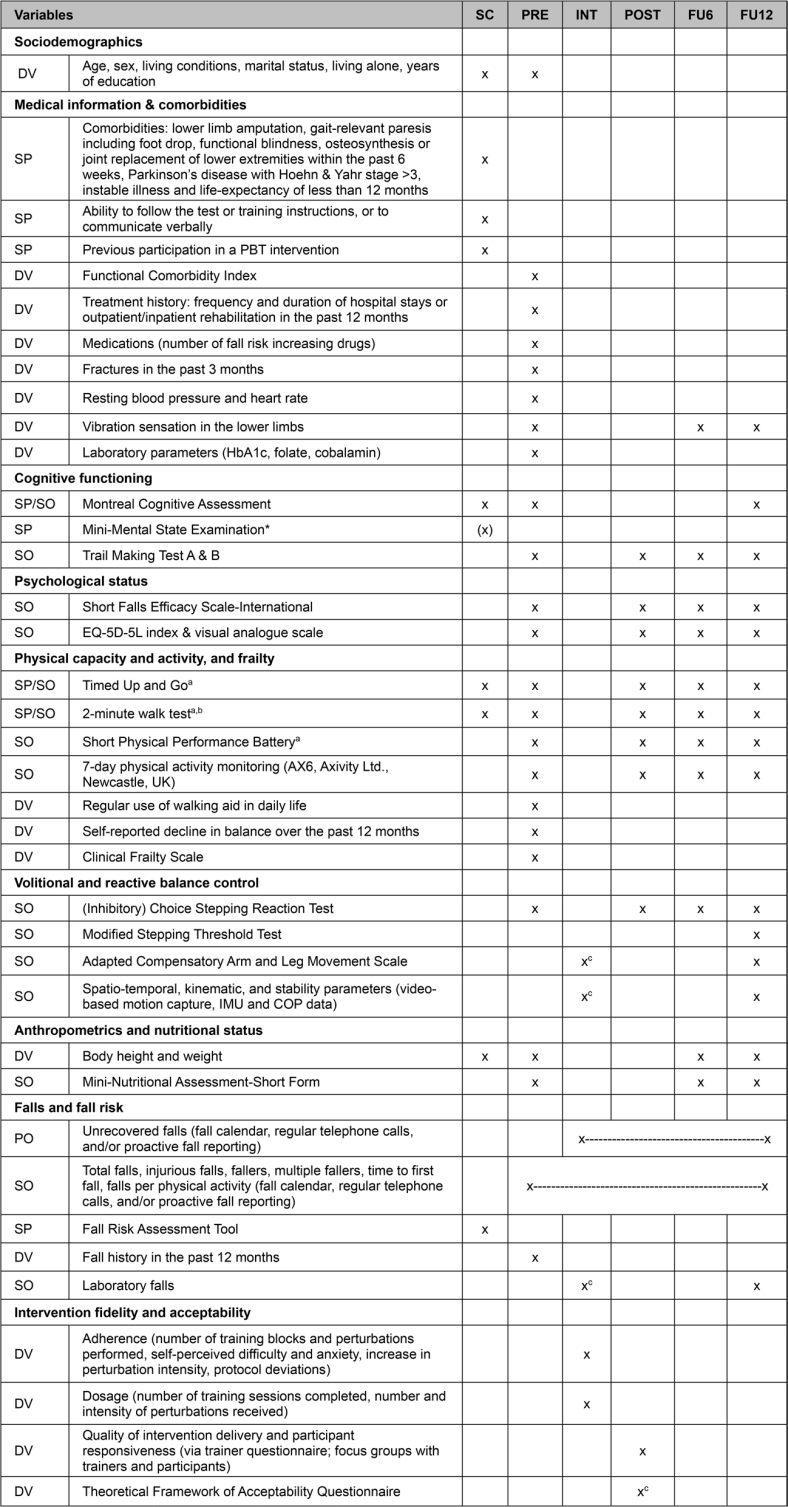
Overview of outcomes measures, screening instruments, and descriptive measures throughout the study timeline

*Abbreviations*: *SC* screening, *PRE* pre-intervention assessment, *INT* within-intervention assessment, *POST* post-intervention assessment, *FU6* 6-month follow-up assessment, *FU12* 12-month follow-up assessment, *PO* primary outcome measure (or part of it), *SO* secondary outcome measure, *DV* descriptive variable, *SP* screening parameter, *PBT* perturbation-based balance training, *IMU* inertial measurement unit, *COP* center of pressure. ^a^instrumented by APDM Mobility Lab; ^b^instrumented by Polar Vantage V3; ^c^only in the intervention group; *Mini-Mental State Examination or Montreal Cognitive Assessment are used for screening eligible participants

#### Primary outcome

The primary outcome is the incidence of unrecovered falls (unrecovered falls per-person years) within 12 months after the end of the intervention. Falls will be recorded continuously through a combination of monthly returned daily fall calendars, monthly telephone calls with participants, and proactive reporting by participants via telephone or email. In addition, proxies are instructed to proactively report any falls experienced by the participant, using the same communication channels. A fall is defined according to the Prevention of Falls Network Europe (ProFaNE) as *‘an unexpected event in which the participant comes to rest on the ground*,* floor*,* or lower level’* [57]. For each reported fall, a structured telephone interview will be conducted covering the circumstances of the fall, time spent on the ground, need for assistance to get up, fall-related injuries and functional limitations, location and environmental factors, pre-fall phase, and potential causes. During this interview, a fall is classified as unrecovered if the participant answers ‘yes’ to the question: ‘*Did someone have to help you get up?*’ [37]. In case of missing, implausible, or contradictory information on unrecovered fall reports from the participant, or if the participant is unavailable for the monthly telephone call (after three contact attempts on different days), the proxy will be contacted to provide (additional) information and/or assess the plausibility of the report using the same structured interview.

#### Secondary outcomes

Key fall-related secondary outcomes will include the number of falls, injurious falls, participants who fall, participants experiencing multiple falls, and time to first fall, as recommended by ProFaNE [57]. The number of falls per physical activity is also assessed to account for risk exposure time for falls [34, 58]. These outcomes will be derived from the same fall reporting strategy described above. Physical activity will be objectively measured over seven days, using a body-fixed sensor (AX6, Axivity Ltd., Newcastle, UK) and validated algorithms [59, 60]. Concerns about falling will be assessed by the Short Falls Efficacy Scale-International, which has been validated in older adults with and without CI [61]. Physical capacity measures will include the Short Physical Performance Battery (SPPB) [62], the TUG [49] and the 2MWT [45, 46], instrumented by inertial measurement units (IMUs; Opal V1, Mobility Lab™, APDM, Inc., Portland, OR, USA) to obtain more detailed movement parameters (e.g., SPPB: postural sway, trunk angle; TUG: sit-to-stand duration, angular velocity of turns; 2MWT: spatio-temporal gait parameters). During the 2MWT, heart rate (Polar Vantage V3, Polar Electro GmbH Deutschland, Büttelborn, Germany) will also be measured to evaluate individual physical effort.

Volitional stepping performance will be assessed with the Choice Stepping Reaction Time Test (CSRT) [63], and the inhibitory CSRT requiring additional response inhibition and response-selection [64].

Static reactive balance will be assessed at FU12 with the modified Stepping Threshold Test (STT) [28, 65], conducted on the BalanceTutor. Single- and multiple-step thresholds - defined as the minimum perturbation magnitudes that trigger one or multiple (≥ 2) recovery steps to regain balance - will be determined based on gradually increasing perturbation intensities. The modified STT used in this study will include an additional initial level with lower intensities to account for more frail individuals. Furthermore, perturbation directions in which a multiple-step threshold is reached remain included in subsequent levels, but at reduced intensity to maintain unpredictability.

Dynamic reactive balance will be assessed at FU12 through observational and kinematic analyses of reactive stepping and laboratory falls in response to eight unannounced submaximal (perturbation intensity level of the BalanceTutor: AP = 15, ML = 10) perturbations (forward, backward, left, right; each during left and right leg swing), applied in random order and at randomized intervals (8–16 s), while walking at an individually preferred speed on the BalanceTutor. This speed will be determined during a 2-min warm-up, following the same procedure as described above for the intervention sessions, but starting at 50% of the overground walking speed measured during the 2MWT at FU12. Additionally, participants will be exposed to one unexpected submaximal forward perturbation (intensity level = 15, swing phase of the left leg) during a 1-min cool-down period, also at the preferred walking speed on the BalanceTutor. The dynamic reactive balance assessment will be video-recorded using a camera (GoPro HERO12 Black, 4 K, 120 Hz, GoPro Inc., San Mateo, California, USA) positioned 59 cm behind the edge of the treadmill platform, perpendicular to the center of the treadmill. The midpoint of the camera lens is set at a height of 103,5 cm from the ground. Data for dynamic reactive balance will be derived from the video recordings, two IMUs (3DT100; MediTouch Ltd., Israel) attached to the participant’s sternum and lumbar region, as well as the center of pressure (COP) data recorded via the BalanceTutor-integrated force plate. Furthermore, participants’ balance recovery strategies in response to the perturbations will be evaluated, applying an adapted version of the Compensatory Arm and Leg Movements (CALM) scale, using video recordings [66]. Video recordings will also be reviewed to determine whether a fall occurred (laboratory fall), defined as a body posture clearly and unambiguously stopped by the BalanceTutor’s safety harness [23].

In the treadmill PBT group, dynamic reactive balance will also be assessed during treadmill PBT sessions three and nine. Participants will receive eight unannounced perturbations (forward, backward, left, right; each during left and right leg swing), applied in random order and at randomized intervals (8–16 s) while walking at the individually preferred speed on the BalanceTutor. The intensity of the AP and ML perturbations will be based on the maximum perturbation intensity achieved during the second treadmill PBT session. Video recordings (CALM, laboratory falls), and IMU and COP data will also be collected during this assessment.

Cognitive functioning will be assessed using the Trail Making Test A and B [67] as well as the MoCA [47].

The 5-level version of the EuroQol-5-Dimension (EQ-5D-5 L) questionnaire and the EuroQol visual analog scale will be used to assess self-reported health-related quality of life and health status [68]).

Acceptability of the treadmill PBT will be assessed in the treadmill PBT group using the Theoretical Framework of Acceptability questionnaire [69].

### Descriptive variables

Age, sex, living conditions, marital status, living situation, and years of education will be collected as sociodemographic information.

Medical information will include comorbidities (Functional Comorbidity Index; [70]), treatments received in the past twelve months, fractures within the past three months, resting blood pressure and heart rate, vibration sensation (Rydel-Seiffer tuning fork), fall risk-increasing drugs, and laboratory parameters (HbA1c, folate, cobalamin), if available from the patient record.

The Mini Nutritional Assessment-Short Form will be used to screen for malnutrition [71].

Frailty status will be assessed using the Clinical Frailty Scale [72]. Self-reported information on the regular use of walking aids in daily life and perceived decline in balance ability over the past 12 months will also be collected.

Intervention fidelity will be measured based on four dimensions [73]: (1) adherence will be quantified based on whether essential components of the intervention session are present or absent, based on a checklist completed by the trainers (i.e., training blocks and perturbations performed, assessment of self-perceived difficulty and anxiety, increase in perturbation intensity, protocol deviations); (2) dosage will be quantified by the number of training sessions completed, and the number and intensity of perturbations received, as automatically recorded by the perturbation treadmill; (3) quality of intervention delivery and (4) participants responsiveness will be measured via a structured questionnaire [69] and focus group interviews of trainers, participants, and health care professionals [74].

### Sample size

The sample size was calculated based on meta-analyses reporting that PBT [75] and gait adaptability training [76], reduce falls by approximately 50% in older adults. For the TRAIL study, it is hypothesized that the intervention group (treadmill PBT) will show a 50% reducion in the incidence of unrecovered falls compared to the control group (CTT). The number of unrecovered falls in the target population is assumed to be 0.5 per person-year [19, 37, 77]. The significance level (α) was set to 5%, and statistical ower at 90%. Sample size calclation was based on a Poisson regression analysis, yielding a required sample size of 132 participants per group to show a significant effect between treatments. The calculation was performed using R 4.0.3 and the WebPower package. Considering an expected dropout rate of 25%, as observed in aprevious intervention study with a similar study population, intervention frequency and duration, and follow-up period [78], sample size increases to 176 participants per group. To account for a potential additional variance due to the multi-center design, the sample size was increased by 10%. Finally, at least 196 participants per treatment group are needed. To obtain equal sample sizes in each group per center, a total of 396 participants (3 × 132 per center) will be recruited.

Losses to follow-up or non-compliance will be included in the intention-to-treat (ITT) analysis.

### Data collection and management

All data will be collected by study nurses and scientific staff. Data will be entered in study-specific electronic case report forms (eCRFs). Furthermore, fall events will be collected using a scannable paper-based fall calendar. Data, registered by the AX6 sensor, the OPAL sensors, the CSRT software, and the Polar watch will be stored on secured servers at each study center. A database constructed using Microsoft Access will be used to extract data in a structured manner, and to organize and standardize the study procedures.

Manuals with standard operating procedures (SOPs) will be used for the conduction of the training intervention as well as the assessments of the study. Prior to the beginning of the study, workshops will ensure standardization, and a similar data quality across all study centers. All study-related measures are documented in these manuals and will be available to all participating partners throughout the study. Data corrections in the eCRF will be documented and are performed by the responsible investigator, or a designated person.

To ensure high quality of data, the completeness, validity and plausibility of data will be checked at the time of data entry (edit-checks), using the database. All data management procedures will be conducted according to written defined SOPs and comply with good clinical practice (GCP). Data will be stored, using pseudonym-codes at the respective study centers first. In frequent intervals Ulm University will collect all data from the study centers, perform plausibility checks, and merge them for statistical analysis.

### Data monitoring and oversight

Risk-based monitoring will be conducted based on the International Council for Harmonization – Good Clinical Practice (ICH-GCP E6) guidelines and SOPs to verify that participants’ rights and wellbeing are protected, reported study data are accurate, complete and verifiable from source documents, and that the study is conducted in compliance with the currently approved protocol/amendment, based on ICH-GCP requirements to ensure safety and integrity of clinical study data. To ensure data accuracy for primary and secondary outcomes, 10% of the source data will undergo verification.

An advisory board consisting of three international experts in the field of PBT in older adults was established to provide scientific oversight, offer feedback, and support the conduct of the study.

### Statistical analysis

The primary outcome, the rate of unrecovered falls within 12 months after the end of the intervention period, will be compared between groups by a multi-level Poisson regression model with study center and randomization block as random effects, at a two-sided significance level of 5%. Analysis of the primary outcome will be done on the full analysis set according to the ITT principle, which will include all randomized participants analyzed in the group they were assigned to. Missing values will be replaced using multiple imputation by chained equations (MICE) with predictive mean matching as imputation method, assuming missing data are missing at random. In total, 10 datasets will be created based on the available data analyzed. Rubin’s rules [79] will be applied to pool results from each dataset. As a sensitivity analysis, a per-protocol analysis will be conducted, consisting of all participants without major protocol violations and with available data on the primary outcome. The intervention effect on secondary outcomes will be analyzed using the same approach as the primary outcome. Additionally, the effect of cognitive status on the primary outcome and key secondary outcomes (total falls, injurious falls, participants who fall, participants experiencing multiple falls, time to first fall, and falls per physical activity, dynamic reactive balance) will be investigated as subgroup analyses. The safety analysis will include calculation of frequencies and rates of (severe) adverse events (AEs) together with 95% confidence intervals on all randomized participants as treated. The analysis will be fully specified in a statistical analysis plan prior to database lock, and will be conducted using R and SAS/STAT^®^ software.

### Harms

AEs will be documented from baseline (pre) until two weeks after the last follow-up (FU12), regardless of any causal relationship to the interventions. During the intervention period and for two weeks following each assessment visit, AEs and serious AEs (SAEs) will be documented in detail. During the remaining study period, only specific risk signals will be recorded: vertebral body fractures, increased osteoarthritis symptoms, activated osteoarthritis or arthritis of the lower extremity, general joint pain (which might indicate arthritis), muscle pain, increase in concerns about falling with impact on (instrumented) activities of daily living or participation in daily life, hospitalization, and mortality.

The severity of AEs is differentiated into grade 1 to 5: mild (grade 1), moderate (grade 2), severe (grade 3), life-threatening (grade 4), death (grade 5). If an (S)AE is assessed as grade 2 or higher, it will be reviewed and verified by medical staff.

All SAEs (grade > 2) during the follow-up period, and AEs (grade > 1) during the intervention period, as described above, will be reviewed and verified regarding causal relationship with the study and their severity classification by an independent data safety monitoring board (DSMB). The DSMB will discuss and decide on changes or discontinuation of the intervention based on the (S)AE reports.

All participants are covered by a subject and travelling accident insurance.

### Patient and public involvement

For ongoing consultation with the target population of the TRAIL study, a participatory research panel (https://uol.de/en/health-services-research/divisions/geriatrie/participatory-health-services-research) is available throughout the study, consisting of patients and healthcare stakeholders. This panel supports the research team during all phases of the study and provides input on the recruitment process, the development of information materials, and the practical implementation of the study. In addition, both the assessments and the intervention were pre-tested with volunteers from the target population to confirm that the study procedures were feasible and acceptable.

### Protocol amendments

The initial recruitment strategy focused on geriatric patients recruited from hospital and inpatient and outpatient rehabilitation settings. To expand the potential recruitment pool, the strategy was extended to the community setting. Additionally, the exclusion criterion for the TUG, used to screen for fall risk, was initially set at < 12 s. According to the recently proposed modifications to the Word Falls Guidelines algorithm for improved fall risk stratification [80], this criterion was revised to TUG < 10 s. These protocol amendments were approved by the local ethics committees at each study site and have also been documented in the trial registry (NCT06652828). Any future substantial amendments to the protocol will be submitted for approval to the ethics committees and documented in the trial registry.

## Discussion

The aim of the TRAIL study is to evaluate the efficacy of treadmill PBT to prevent unrecovered falls in fall-prone older adults with and without CI. To the best of our knowledge, this is the first multi-center randomized controlled trial specifically targeting a high-risk population for falls, with unrecovered falls as the primary outcome.

In previous studies on the efficacy of PBT for fall prevention, potential ceiling effects have been suggested, largely attributed to the inclusion of healthier, community-dwelling older adults with a lower risk of falling [30]. To address this limitation, older adults who complete the TUG in less than 10 s without the use of a walking aid will be excluded from this study [80], thereby ensuring a focus on individuals at higher risk of falls.

Importantly, individuals with moderate CI will be included in this study – a group that remains often underrepresented in fall prevention research [21]. To date, no study has specifically investigated whether PBT is equally effective and demonstrates similar retention in older adults with and without CI. The low training volume of treadmill PBT, which is expected to be sufficient to achieve a significant reduction in falls [24], may be particularly relevant for individuals with CI, for whom longer training periods are less likely to be accomplished. The treadmill PBT intervention was designed to closely resemble typical physiotherapy prescription practices within the German health care system, thereby facilitating its integration into routine care, if proven effective.

The sample size calculation was based on an anticipated 50% reduction in falls, informed by meta-analyses in older adults on reactive and volitional stepping interventions by Okubo et al. [75] and gait adaptability training by Nørgaard et al. [76]. PBT can be conceptualized as a specific form of gait adaptability training, as it challenges the ability to respond to sudden changes during walking. Therefore, the findings by Nørgaard et al. [76] were also considered relevant to the expected effect of the treadmill PBT intervention. This assumption has since been supported—though at a somewhat lower effect size—by a more recent meta-analysis by Devasahayam et al. [22], which reported a 39% reduction in alls through reactive balance training.

For fall reporting in older adults, prospective daily fall calendars with at least monthly reporting, followed by telephone calls to clarify missing data and gather additional details on falls and injuries, have been recommended [57]. Following the recommendations by Zieschang et al. [34] for fall reporting in older adults with CI, the TRAIL study will use a combined strategy of monthly returned daily fall calendars, regular once-monthly telephone calls, and proxy information to provide additional context or assess the plausibility of participant-reported falls in cases of missing, implausible, or contradictory information. The TRAIL study further extends these sources of information by encouraging participants and proxies to proactively report falls, which may enhance the completeness of fall data and support greater participant engagement in the reporting process.

PBT triggers automatic reflex-like balance responses that help to restore equilibrium without the need for conscious cognitive processing. This feature may be particularly beneficial for individuals with CI. In addition to its promising low volume, treadmill PBT is perceived as effective by both older adults and health care professionals [81, 82]. However, these positive perceptions must still be validated in populations with CI.

Reactive balance assessment will be conducted in both groups only at the 12-month follow-up, based on two considerations. First, such assessment could unintentionally influence reactive balance and fall outcomes in the control group, potentially confounding the effects of the PBT intervention. Second, exposing participants to their limits of reactive balance control already at baseline might cause anxiety about upcoming perturbations during the intervention period, which may lead to early dropouts [28]. However, given a successful randomization of participants, we assume a comparable level of reactive balance control in both groups at baseline. Therefore, if training effects are retained, differences in reactive balance may become evident at the 12-month follow-up.

The potential public health impact of treadmill PBT is considerable. The intervention could be implemented into existing health care structures such as day clinics, inpatient and outpatient physiotherapy settings, rehabilitation centers, or even fitness centers. Treadmill PBT offers a safe, task-specific approach to fall prevention, with a minimal risk of injury due to the use of a safety harness system. As such, it holds significant promise for improving care for the rapidly growing population of fall-prone older adults.

From an economic viewpoint, falls in older people pose a significant economic burden to society, accounting for 0.9% to 1.5% of total health care expenditures related to falls alone. In Germany, the costs for treating fall-related injuries exceeds 2 billion Euros, annually [83]. Treadmill PBT may be more cost-effective compared to conventional physical exercise. The minimal set up time of the perturbation treadmill (< 1 min) and the low training volume of treadmill PBT are important features for future implementation into health care systems and for broader scalability. However, the initial acquisition costs of the treadmill need to be considered, and the overall economic efficiency warrants further investigation.

## Conclusions

As one of the first treadmill PBT studies targeting a fall-prone older population, also including individuals with CI, the TRAIL study addresses key limitations of previous PBT research, which has predominantly involved healthier older adults. With only a limited training volume, treadmill PBT is expected to provide a high benefit for participants, including a meaningful reduction in falls in combination with functional improvements. Notably, these benefits may also extend to participants with CI – a population for whom effective fall prevention strategies are limited. Overall, the TRAIL study will provide important evidence on the clinical and public health value of treadmill PBT for fall prevention in a vulnerable population.

## Trial status

Recruitment for the TRAIL study started in November 2024. The current protocol version is 4.0, with the last amendment on March 25th, 2025. At the time of submission (28.07.2025), 158 participants have already been enrolled in the study.

## Supplementary Information

Supplementary Material 1.

Supplementary Material 2.

## Data Availability

No datasets were generated or analysed during the current study.

## Abbreviations

2MWT: 2-minute walk test
AP: anterior-posterior
CALM: Compensatory Arm and Leg Movements
CI: cognitive impairment
COP: center of pressure
CSRT: Choice Stepping Reaction Time
CTT: conventional treadmill training
FU6: 6-month follow-up assessment
FU12: 12-month follow-up assessment
IMU: inertial measurement unit
ML: mediolateral
MMSE: Mini-Mental State Examination
MoCA: Montreal Cognitive Assessment
PBT: perturbation-based balance training
Post: post-training assessment
Pre: baseline assessment
ProFaNE: Prevention of Falls Network Europe
SPPB: Short Physical Performance Battery
STT: Stepping Threshold Test
TIDieR: Template for Intervention Description and Replication
TUG: Timed Up and Go
