# Supplementary material for: Treadmill perturbation-based balance training to prevent unrecovered falls in fall-prone older adults with and without cognitive impairment: protocol for the multi-center randomized controlled TRAIL study
Source: BMC Geriatr. 2025 Dec 23;26:143. doi: 10.1186/s12877-025-06599-w (PMC12860074; doi:10.1186/s12877-025-06599-w)
Supplement: Supplementary file 2 — Supplementary Material 2. [file 12877_2025_6599_MOESM2_ESM.docx]

**Supplementary Table 1** Description of the interventions using the Template for Intervention Description and Replication (TIDieR) checklist

| **Item** | **Description** |
| --- | --- |
| 1. Brief name | Treadmill perturbation-based balance training (PBT, intervention) and conventional treadmill training (CTT, control) |
| 2. Why | Treadmill PBT is typically applied to low-risk older adults, despite fall-related injuries being most prevalent among individuals at high risk of falling. To date, treadmill PBT has not been systematically evaluated in high-risk populations for falls, and no clinical trials have investigated whether treadmill PBT is equally effective or exhibits similar retention patterns in older adults with and without cognitive impairment. |
| 3. What:  Equipment | Treadmill PBT  Delivered on a computer-controlled perturbation treadmill with a safety harness system (BalanceTutor, MediTouch Ltd., Netanya, Israel) that allows for backward (belt acceleration), forward (belt deceleration), left and right (platform shift) surface perturbations with 30 different levels of magnitude.    CTT  Administered using also the BalanceTutor, functioning solely as a computer-controlled treadmill with a safety harness. During the CTT, no perturbations will be applied; the treadmill will be used exclusively for standard walking. |
| 4. What:  Procedures | Treadmill PBT  Nine sessions on the BalanceTutor supervised by an instructed trainer. Each session starts with 2.5 minutes of unperturbed walking to warm up and determine the individually preferred walking speed. This is determined by starting at 50% of the participant’s habitual overground walking speed (from the baseline 2-minute walk test), then gradually increasing and decreasing treadmill speed to identify the upper and lower bounds of comfortable walking speed. The average speed of these bounds is defined as the preferred walking speed and used for all training blocks in that session.  Session 1 includes four blocks of perturbed walking, whereas sessions 2 to 9 each comprise five blocks of perturbed walking. Each block lasts about 2.5 minutes followed by a 1-2-min break. Within the breaks, the trainer assesses the participant’s perceived difficulty and anxiety via a 5-point Likert scale. In addition, the trainer assesses how difficult the training was for the participant via a 5-point Likert scale (1 = easy, 2 = fairly easy, 3 = challenging, 4 = very challenging, 5 = too hard) to adapt the perturbation magnitude in the subsequent block. Over the nine sessions, there is a progression through an increased number and magnitude of perturbations as well as a reduced degree of predictability of the time, direction, and strength of the perturbation. Perturbations are administered within a randomized interval of 7 to 15 seconds following the preceding perturbation, and are timed to coincide with the swing phase of either the left or right leg.   - Session 1: One block of unperturbed walking (block 1). Four blocks of perturbed walking in backward (block 2-3) and forward (block 4-5) direction. Six perturbations for each block (total 24 perturbations). Predictable timing and direction. Trainer-rated level of difficulty ≤3 (5-point Likert scale). - Session 2: Five blocks of perturbed walking in right (block 1-2), left (block 3-4) and right/left/forward/backward direction (block 5). Block 1-4: Six perturbations for each block. Block 5: Eight perturbations (total 32 perturbations). Predictable timing and direction. Trainer-rated level of difficulty ≤3. - Session 3: Five blocks of perturbed walking. Block 1: Dynamic reactive balance assessment*. Block 2 in the forwards/backwards direction. Block 3 in left/right direction. Block 4-5 in all directions. Eight perturbations for each block (total 40 perturbations). Predictable direction. Trainer-rated level of difficulty = 3-4. - Session 4-5: Five blocks of perturbed walking in all directions. Eight perturbations for each block (total 40 perturbations). Non-predictable. Trainer-rated level of difficulty: 3-4. - Session 6: Five blocks of perturbed walking in all directions. Eight perturbations for each block (total 40 perturbations). Non-predictable. Trainer-rated level of difficulty varies between blocks: high 3-4, low 2-3. - Session 7: Five blocks of perturbed walking in all directions. Eight perturbations for each block (total 40 perturbations). Non-predictable. Trainer-rated level of difficulty varies between blocks and perturbations: high 3-4, low 2-3. - Session 8: Identical to session 6 but with the addition of watching videos during training. - Session 9: Identical to session 7 but with the addition of watching videos during training. Block 1: Dynamic reactive balance assessment*     CTT  Nine CTT sessions on BalanceTutor supervised by an instructed trainer. Each session starts with 2.5-min unperturbed walking to warm-up and identify individually preferred walking speed. Sessions 1–9 consist of five blocks of conventional treadmill walking, each lasting 2.5 minutes, followed by a 1-2-min break. |
| 5. Who provided | Treadmill PBT and CTT will be administered by trainers (sports scientists and physiotherapists) who have undergone extensive instruction on operating the treadmills and who deliver the training based on a standardized trainer manual and detailed guidelines to ensure fidelity of the intervention delivery. |
| 6. How | Treadmill PBT and CTT are delivered in a one-to-one format, with each participant receiving individualized guidance and supervision from a dedicated trainer. |
| 7. Where | The study is conducted across three sites: Geriatric Center, Medical Faculty Heidelberg, Heidelberg University; Marien Hospital Herne – University Hospital Ruhr-University Bochum; and University of Oldenburg, Geriatric Medicine, Oldenburg, Germany |
| 8. When and how much | Both treadmill PBT and CTT are delivered over nine 30-min sessions, scheduled three times per week over a 3-week period. In case of illness, scheduling conflicts or organizational issues, the training period may be extended to a maximum of six weeks to ensure completion of all nine sessions. |
| 9. Tailoring | Perturbation magnitude (treadmill PBT): Individually tailored according to the trainer-assessed level of difficulty, rated using a 5-point Likert scale.  Treadmill speed (treadmill PBT and CTT): Initially (session 1) determined by each participant’s habitual overground walking speed, assessed during the baseline assessment, and then gradually adjusted during the 2.5-min warm-up based on participant feedback to achieve the individually preferred walking speed. In subsequent sessions, the individually preferred walking speed will again be determined during the 2.5-min warm-up, using the walking speed from the previous session as a starting point. |
| 10. Modifications | N/A |
| 11. How well:  Planned | Training adherence to both treadmill PBT and CTT sessions will be documented by the trainers. For all treadmill PBT sessions, the number and magnitude of applied perturbations will be automatically documented by the BalanceTutor software. |
| 12. How well:  Actual | N/A |
| *Participants will receive eight unannounced perturbations (forward, backward, left, right; each during left and right leg swing), applied in random order and at randomized intervals (8-16 seconds) while walking at the individually preferred speed on the BalanceTutor. The intensity of the perturbations will be based on the maximum perturbation intensity achieved during the treadmill PBT session 2, block 5. | |
